# ‘Such a massive part of rehab is between the ears’; barriers to and facilitators of anterior cruciate ligament reconstruction rehabilitation: a qualitative focus group analysis

**DOI:** 10.1186/s13102-022-00499-x

**Published:** 2022-06-15

**Authors:** Adam Walker, Wayne Hing, Suzanne Gough, Anna Lorimer

**Affiliations:** 1 Faculty of Health Sciences and Medicine, Bond University, Gold Coast, 4226 Australia; 2 Bond Institute of Health and Sport, Promethean Way, Robina, QLD 4226 Australia

**Keywords:** Physiotherapy, Return to sport, Adherence, Compliance

## Abstract

**Background:**

Current evidence demonstrates that few patients complete anterior cruciate ligament reconstruction rehabilitation according to evidence-based guidelines. It is important to investigate the viewpoints of our patients to identify patient-reported barriers and facilitators of anterior cruciate ligament reconstruction rehabilitation. Qualitative analysis can provide insight into potential methods for improving the delivery of rehabilitation services.

**Methods:**

In this qualitative study, utilising a social constructionism orientation, viewed through the social phenomenological lens, three focus groups were conducted with individuals 1–20 years post anterior cruciate ligament reconstruction (n = 20, 9 males, 11 females, mean 6.5 years post-surgery, 19–51 years old). Utilising a semi-structured interview guide, participants were asked about their experiences during anterior cruciate ligament reconstruction rehabilitation. Focus groups were recorded, transcribed, and coded using an inductive semantic thematic analysis methodology.

**Results:**

Five organising themes were identified (consisting of 19 sub-themes) to provide a framework to present the data: psychological, physiological, rehabilitation service, rehabilitation characteristics, and interaction with others. Each theme details aspects of rehabilitation, such as exercise delivery, informational support, frequency, and duration of care, kinesiophobia, weight management and interactions with teams and coaches, which present barriers or facilitators for patients to adhere to and participate in rehabilitation. Example quotes are provided for each theme to provide context and the patient’s voice.

**Conclusions:**

This qualitative investigation identified key aspects of a patient's rehabilitation in which they encounter a variety of barriers and facilitators of ACL reconstruction rehabilitation. These aspects, such as the rehabilitation characteristics, service delivery, psychological and physiological factors, and interactions with others, were consistently identified by this cohort as factors which affected their rehabilitation. The themes may provide targets for clinicians to improve rehabilitation and deliver patient-centred care. However, the themes must be evaluated in future trials to assess whether interventions to remove barriers or enhance facilitators improves subsequent outcomes such as return to sport and re-injury rates.

**Supplementary Information:**

The online version contains supplementary material available at 10.1186/s13102-022-00499-x.

## Background

A typical anterior cruciate ligament (ACL) reconstruction rehabilitation lasts approximately 12 months before return to sport [1]. Recent observational studies found that those who completed more thorough rehabilitation beyond 6 months including structured gym, agility, and landing tasks are less likely to have not returned to ‘high risk’ sport and those who had were more likely to pass return to sport testing criteria [2, 3]. Current evidence suggests that only 30% of patients complete evidence-based rehabilitation beyond six months, and less than 5% will complete rehabilitation according to current evidence-based guidelines [4]. There are likely many reasons why patients fail to participate in and adhere to evidence-based recommendations [5].

As a result, it is important to seek the viewpoints and investigate the experiences of our patients ACL reconstruction rehabilitation to gain insight into potential methods for improving the delivery of rehabilitation services. Previous research has provided evidence on the key role the treating health practitioner plays in setting an appropriate rehabilitation environment to reduce treatment-related barriers and enhance rehabilitation facilitators [5]. Through optimising rehabilitation, potentially many of the barriers to return to sport, namely poor physical function and kinesiophobia [6, 7], could be overcome.

Qualitative methodologies, such as focus group analyses, are an appropriate methodology to investigate patients’ barriers to and facilitators of rehabilitation. Qualitative methodologies enable a more complete picture of a person's experience than quantitative research alone [8]. Whilst there is some qualitative research into patients experience of rehabilitation interventions and return to sport, there is a lack of research investigating the barriers to and facilitators of rehabilitation participation [5]. This study is part of a series of research and a direct follow up of previous survey research [9]. The survey was guided by the results of a scoping review on this topic [5]. The objective of this research is to investigate the patient-reported barriers and facilitators of ACL reconstruction rehabilitation. With this, we aim to identify targets to facilitate participation and adherence to rehabilitation.

## Methods

### Theoretical and methodological approach

This qualitative study utilised a social constructionism orientation and is viewed through the social phenomenological lens [10]. Constructionism believes that all knowledge and meaningful reality is contingent upon human practices and interactions between human beings and their world within a social context, namely the interaction between the rehabilitation provider, patient, rehabilitation environment and health care system [10]. We aimed to report on the experiences, meanings and reality of participants, focusing on illuminating the details within the rehabilitation experience that may be missed or poorly understood to create meaning and achieve a sense of understanding [11, 12]. The methodology was developed to enhance the rigour and trustworthiness of the data and reporting per the Consolidated Criteria for Reporting Qualitative Research. The research received approval from the Bond University Human Research Ethics Committee (AW02850).

### Participants

At the end of the survey [9], participants consented to be contacted for participation in a focus group interview. Inclusion criteria were: (1) aged 18 years or older, (2) prior ACL reconstruction in the past 20 years, as rehabilitation practices were deemed to have significantly changed before 2000 [13]. Any graft type and concomitant injury were eligible as they may provide further context to an individual’s rehabilitation experience.

### Data collection

Forty-two potential participants were contacted via email, with 20 consenting to participate in the focus group. Participants completed an online survey to collect personal, injury, sport, and demographic information. Participants were then allocated into one of three focus groups, based on availability and personal demographics, to create heterogeneous groups based on age, gender, injury, sport level and type to facilitate variations in personal experiences.

Each focus group was allocated 6–8 participants and was estimated to last no longer than 60 min. Each participant was provided with an explanatory statement and signed a consent form informing them of the purpose, requirements, ethical considerations, and their rights in undertaking the research. The project lead (AW) conducted the focus groups at the University guided by a semi-structured interview guide (Additional file 1) mapped to the research questions. The guide was piloted with a sample participant to ensure understanding, focusing on question style and structure. No modifications were made. The pilot data was not included in the analysis. One researcher (WH or AL) acted as a note-taker during the focus groups. Once the research team deemed that the interview had reached theoretical saturation, the interview ceased. No new themes emerged following the third focus group indicating that data saturation had occurred, and no further focus group sessions were required.

### Data analysis

The analysis utilised an inductive semantic thematic analysis method, guided by the six-step process outlined by Braun and Clarke [12]. First, the audio recording of the focus groups was transcribed verbatim and verified by the authors using a transcription convention on the secure transcription service ‘Rev’ (rev.com/transcription). The transcriptions were not returned to the participants for review. Then the data was evaluated and coded using the qualitative data analysis software NVivo Version 12 (QSR International Pty. Ltd) by identifying initial codes, searching for themes and revising and defining the themes [12]. Identifying and forming a thematic framework drew upon existing a-priori issues [5], topics introduced in the interview guide, emergent issues raised by participants themselves, and analytical themes arising from the recurrence or patterning of particular views or experiences [12].

One member of the research team completed the analysis independently coding to identify themes and subthemes: an experienced physiotherapist within ACL reconstruction rehabilitation and completing a pragmatic mixed methods research project into ACL reconstruction rehabilitation. The authorship team had a diverse and extensive range of experience and perspectives within musculoskeletal research. One research team member (SG) has extensive knowledge and experience within qualitative research methodology and thematic analysis. All authors assisted in reviewing and defining themes to check for consistency and stability and producing the report.

## Results

We conducted three focus groups with six or seven participants, with a total of 20 individuals 1–20 years (mean 6.5 years) post ACL reconstruction. Focus groups lasted between 65 and 72 min. Each group consisted of a mix of genders (9 males, 11 females), age ranges (19–51), sports, concomitant injuries, and participation levels. Twenty-five percent of participants had returned to their previous level of participation, and 85% reported having ongoing problems with their knee. All participants returned to a lower level of competition, changed, or did not return to sport due to factors relating to their knee, except participants 16 and 19 who returned to a lower level of competition due to other factors not related to their injury. When asked about their return to sport, participants reported that fear of movement (kinesiophobia) or reinjury and ongoing symptoms were the primary factors that affected their ability to return to their previous level of sport. Details of the selected participants are presented in Table 1.

**Table 1:** Participant details

Participant	Age	Sex	Second injury	Time since injury (years)	Associated injuries	Main sport	Level of sport	Time to RTS (months)	Level of sport competition returned to	Ongoing problems
1	51	M		4	Unsure	Soccer	Local	18	Changed sports	None
2	50	F		4	Unsure	Netball	Social	24	Lower level	Minor
3	21	M		2	Meniscus, LCL	Touch Football	International	13	Changed sports	None
4	47	M	Y	12	Meniscus	Touch Football	National	9	Previous or higher level	Minor
5	29	F		13	None	Soccer	Local	12	Changed sports	None
6	29	M		1	PCL	Cricket	Local	13	Lower level	Moderate
7	45	F	Y	20	Meniscus, MCL, cartilage	Touch	National	17	Lower level	Minor
8	48	F		2	MCL	Tennis	Local	24	Changed sports	Moderate
9	38	M		18	Meniscus	Rugby	Regional	7	Lower level	Significant
10	24	F		2	MCL	Netball	Local	12	Previous or higher level	Minor
11	50	F		5	Meniscus	Gym/running	Social	-	Did not return to any sport	Minor
12	27	M		3	None	Netball	National	9	Previous or higher level	Minor
13	31	M	Y	11	Meniscus	Soccer	National	7	Previous or higher level	Significant
14	28	F		4	Meniscus, MCL, LCL	Weightlifting	Social	24	Did not return to any sports	Minor
15	19	M		2	Meniscus, fracture	Cricket	Regional	10	Previous or higher level	Minor
16	28	M		7	Meniscus, MCL	Rugby Union	Local	12	Lower level	Minor
17	40	F		2	Meniscus	Netball	Social	10	Changed sports	Minor
18	22	F	Y	6	Meniscus, MCL	Volleyball	Regional	24	Lower level	Minor
19	46	F		2	MCL, cartilage	Hockey	Social	12	Lower level	Minor
20	28	F		11	None	Netball	Regional	15	Lower level	Minor

M, male; F, female; Y, yes; MCL, medial collateral ligament; LCL, lateral collateral ligament; PCL, posterior cruciate ligament; RTS, return to sport

**Fig. 1:**
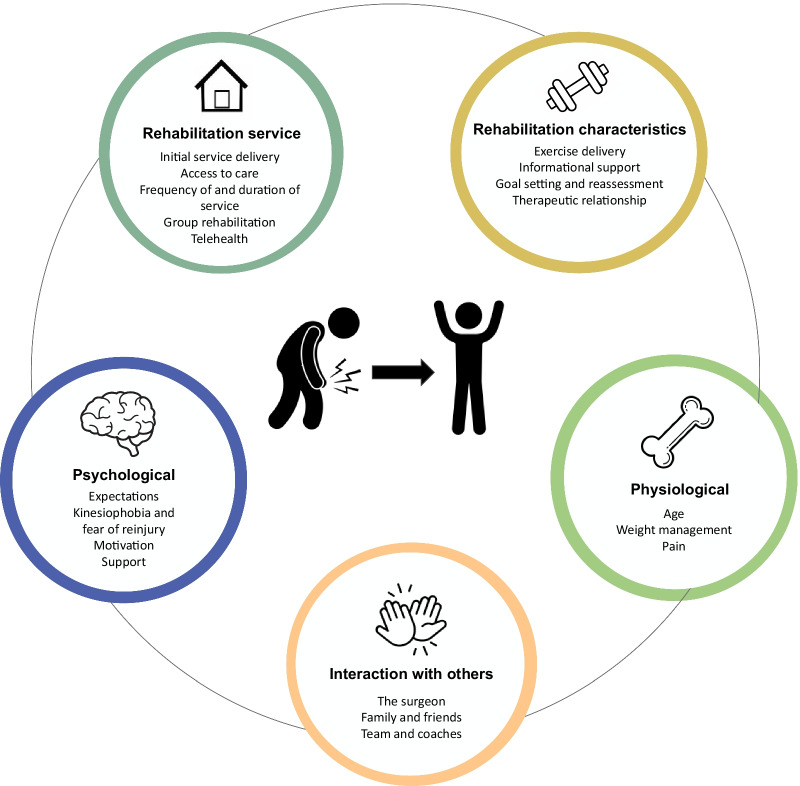
The five organising themes influence the patient’s rehabilitation journey from injury to return to sport (original work by authors)

Five organising themes were identified as key aspects of rehabilitation that present barriers and facilitators of rehabilitation adherence and participation on a patient’s journey back to sport. The research team developed a visual framework representing each theme as influencing factors of a patient's rehabilitation journey to return to sport (Fig. 1). The organising themes and associated sub-themes are not weighted, as each participant experiences barriers and facilitators differently according to their rehabilitation journey. In no order, the organising themes are: (1) psychological, (2) physiological, (3) the rehabilitation service, (4) the rehabilitation characteristics and (5) interactions with others. Nineteen sub-themes associated with each organising theme were formed based on the line-by-line coding of the transcript. Additional file two provides additional verbatim quotations for each subtheme.

### Organising theme 1: Psychological factors

Participants detailed psychological factors which impacted their rehabilitation. Four subthemes were identified: (1) the participant's expectations for rehabilitation, (2) the impact of kinesiophobia and fear of re-injury on rehabilitation, (3) the difficulties in staying motivated throughout rehabilitation, and (4) the need for support from clinicians, friends, family, their team, and external sources.

#### Expectations

Most participants expected a 12-month recovery but underestimated the effort required in completing rehabilitation to a sufficient standard and intensity to address post-operative deficits. They felt health practitioners failed to inform them of the possibility of ongoing pain and other injuries. However, they believed that physiotherapists are in the best position to set realistic expectations with their patients due to the ongoing relationship and early input (ideally before surgery). As explained by one participant, "If they were to see a physio beforehand, maybe they would have an understanding of what you've got to do here and that it's going to be a longer journey because if those expectations are, I guess, worked out beforehand, you're less likely to have that disappointment or that depression and things that go on after surgery” (participant 20).

#### Kinesiophobia and fear of re-injury

Participants consistently reported that exposure to situations associated with their injury, such as jumping and change of direction, were the hardest to overcome. However, repetition and controlled exposure to these situations aided their ability to overcome their fears. As explained, “just making it more sport-specific, just recreating some of those scenarios that are scary. So, make sure you're comfortable with what you're doing and relate it into your sport” (participant 7).

#### Motivation

Some participants lost motivation to complete rehabilitation due to not feeling supported in their return to sport ambitions. Slow progression and other priorities through the mid-stages of rehabilitation made it challenging to maintain motivation in completing rehabilitation; however, striving for and achieving key milestones and goals assisted in maintaining motivation over the long term. For example, "that one stage where it's like, yes, you can run for as long as you can. That was super … It was motivating” (participant 15). Exposure to athletes who had returned to high-level sport also increased motivation.

#### Support

Having a supportive team to guide you through surgery, recovery and rehabilitation aided in achieving a successful outcome. Support could be from external sources, such as online support groups, friends and family or the treating health practitioners. As explained by one participant, "one thing that's come out of just hearing everything today is that such a massive part of rehab is between the ears… that mental side and it all starts from the minute you walk into the door to your surgeon, and then you pick your physio and that person, and how they understand what you are going through” (participant 1).

### Organising theme 2: Physiological factors

Participants mentioned physiological factors which impacted their rehabilitation; three subthemes were identified. These are: (1) older individuals feeling discriminated against, (2) post-operative weight management, and (3) the challenge of pain during rehabilitation.

#### Age

Unlike younger individuals, older individuals felt surgeons and physiotherapists failed to consider their return to sport goals. As detailed by one participant, “he must have said five, six times, well, at your age, women your age tend to. Do you want to just do Pilates? Do you want to just walk on the beach, because if that's the case, you don't need your ACL done? And I felt very patronised" (participant 2). Older individuals also felt it was slower and harder to recover from the surgery.

#### Weight gain

Participants were seeking alternative means to exercise and dietary information to adjust to post-surgical inactivity. Many participants reported weight gain hindered rehabilitation and failed to lose it long term. As suggested by one participant, “I think a nutrition plan will help as well. Like you said, gaining weight is a potential issue…. And you're just like, I don't even know how to eat at all. I think that is really important” (participant 13).

#### Pain

Participants reported persistent pain and complications such as patellofemoral pain, delayed rehabilitation progression and reduced adherence. "The fear, for me, was that I couldn't get rid of kneecap pain for so long and whether that was ever going to go away” (participant 19).

### Organising theme 3: Rehabilitation service

Factors related to the delivery of the rehabilitation service were identified, resulting in five subthemes. These are: (1) challenges during their initial utilisation of health services, (2) difficulties in accessing health care, (3) the factors which influence the frequency and duration of supervised rehabilitation and (4) the utility of group rehabilitation and (5) telehealth as modes of service delivery.

#### Initial service delivery

Initial interactions within the health care system were reported to present barriers to rehabilitation progression through delays in diagnosis and a failure of first contact practitioners to hear patient concerns. Many participants had similar stories, “I found it was kind of disconcerting when everyone's going around sharing their ACL stories that like half of us, when we were initially diagnosed was saying, oh, it's just bone bruising, like, oh, it's just a sprain." (participant 15). Participants also had a desire for a plan that encompassed a variety of options for their care to prepare for the entirety of rehabilitation adequately. Remarkably few participants completed any form of prehabilitation, but it was highly valued in those who did.

#### Access to health care

Having the time to travel to and attend appointments was a common barrier for participants. Access issues were particularly evident in the early phases due to post-operative driving restrictions and others with high family and work commitments. “I think probably the difficulty for me was being a mum of two, working full time…. That was my difficulty” (participant 17).

#### Frequency and duration of service

As rehabilitation progressed, the ever-increasing cost became a burden to participants and may result in early cessation of care; "So, I think the biggest barrier is access to physio being more affordable in Australia…. I'm at the start of my journey, that could be quite expensive over nine months for me to afford” (participant 6). Participants who ceased rehabilitation early (before six months) reported wishing they had continued longer to avoid long term problems. Some participants reported periodic review in the later stages of rehabilitation, at the recommendation of the physiotherapist, made it difficult to maintain motivation and overcome physical impairments, build confidence and physical capacities for return to sport.

#### Group rehabilitation

Group rehabilitation was widely supported across participants to facilitate rehabilitation progression by developing physical capacities while also providing support and motivation through interaction with others. One patient noted, "Just to be part of a group of people that we had gone through the same thing, just for accountability and motivation because you lose your self-confidence until you can really get going again” (participant 8).

#### Telehealth

Telehealth was accepted as a mode of service to reduce financial and travel burdens but only in combination with in-person appointments to monitor pain, exercise technique, overcome kinesiophobia and deliver hands-on therapies. As one patient expressed, “I think there're some things that you could've done fine with telehealth that probably would've helped with the cost of some of it and the access…. but like some of the stuff that I had to do with my physio, I don't think I would ever have been able to do it if I didn't feel they were right there to catch me if I fell off a block. I just wouldn't have done it. I don't think” (p*articipant 10).*

### Organising theme 4: Rehabilitation characteristics

Participants spoke about barriers and facilitators they encountered during rehabilitation sessions and interactions with their physiotherapist. The four subthemes identified were: (1) difficulties in completing exercises independently, (2) the desire for informational support, (3) the need for clear and progressive goal setting and reassessment and (4) the relationship with their physiotherapist built on trust and collaboration.

#### Exercise delivery

Participants consistently reported difficulty completing their rehabilitation as prescribed due to therapists providing unclear instructions and excessive use of technical language. As one participant described, “I probably didn't feel like I was getting very good instruction as to what to do… It wasn't that I wasn't willing to commit to the time, it was I just wasn't sure what I was supposed to be doing” (participant 19). However, participants highly valued being informed of the reason for exercise selection and a clear path for progression. To enhance exercise adherence, they recommended using exercise prescription applications and the therapist considering individual circumstances such as work, family, and exercise competency. Further, participants often expressed that the late stage of rehabilitation was poorly executed and failed to expose them to training to prepare them for a return to sport; “I think the other barrier for physio is once you get to nine months, and you're doing most of your strengths pretty equal, is that specific skills that you want to be outside running, changing direction, and you can't do that in a small clinic, and that's kind of where I left once, I wanted to be outside” (participant 7).

#### Informational support

Due to the long rehabilitation process, participants consistently reported seeking external sources of information to assess their progress, answer questions and provide motivation. They did express concern over verifying external sources of information. They recommended therapists “not give all of the information the first time you see someone, because it's just too much. It's like a drip-feed, and then building on it each time” (participant 5).

#### Goal setting and reassessment

Participants highly valued the process of collaboratively setting and achieving relevant goals and milestones but reported that it was poorly executed (e.g., solely time-based) or never provided to them; "’Here's where you are. This is where you're going. These are our checkpoints along the way.’ And then let's talk about it, and see if you're happy with it, and if we're there and if not, why not? What are we going to do about it to get you to where you should be? I mean, that didn't happen to me, and I thought it should've done” (participant 1). Setting non-clinical goals (such as completing fun runs or fitness challenges) should also be considered.

#### Therapeutic relationship

Many participants described how a strong therapeutic relationship built on trust, knowledge and support enhanced rehabilitation and guided them through a lengthy rehabilitation process. For example, one participant stated, “it's the relationship that you build which is based on trust. You trust that that physio will get you there, and the physio has also got to trust that you're going to do your job, and you're not going to let the physio down. So, it's building that” (participant 2). They also expressed that the selection of the wrong therapist can significantly hinder rehabilitation.

### Organising theme 5: Interactions with others

Participants spoke on how their interactions with others or the wider community impacted their rehabilitation. Three subthemes were identified: (1) the ability of the surgeon to influence the plan for care and rehabilitation, (2) the importance of supportive family and friends and (3) the desire to stay involved with their team.

#### The surgeon

Some participants reported a positive, motivating, and supportive interaction enabled rehabilitation; however, some initial surgeon interactions were perceived as impersonal, negative, and demotivating, which inhibited recovery. The surgeon is in a highly influential position, “whatever they say, you believe because…. you put your trust in them…. they need to build some comfort and warmth inside, warm and fuzzy to make you feel that it's going to be okay” (participant 1). Participants also reported never being recommended to undertake a period of prehabilitation from their surgeon.

#### Friends and family

Participants valued friends and family who facilitated rehabilitation through offering transport, encouragement, and supervision during rehabilitation. However, they often felt that others failed to understand the significance of the injury; “I think people around you didn't understand either, isn't it? Because it's like when you hurt your back because it's not a big gaping wound and people can't see it, they don't… Not as much empathy” (participant 11).

#### Team and coaches

The opportunity to stay involved with their team through coaching and support roles was highly valued by participants. They often felt isolated from their team when unable to participate in training and games. As one participant detailed, “I was a little bit isolated. Because the team, they're doing a real intense workout and you're like, I'm just going to use some TheraBand’s and hope for the best” (participant 10).

## Discussion

The current study investigated the patient-reported barriers and facilitators of ACL reconstruction rehabilitation. The five organising themes identified and associated subthemes provide the clinician with a patient’s perspective of the barriers encountered as patients pursue their activity and sport goals. The specific themes, contexts and examples identified during this qualitative investigation provide targets for clinicians to improve the rehabilitation experience and facilitate adherence to evidence-based rehabilitation.

### Psychological

Patients’ psychological response to injury, surgery and ongoing rehabilitation is heavily influenced by the practitioners involved in their care. Patients typically have high expectations for returning to sport that is often not met, highlighting the importance of setting clear expectations early in rehabilitation [14], not only about rehabilitation milestones but also regarding time commitment, cost, frequency and duration of rehabilitation. Furthermore, participants wanted to be informed on the likely outcomes for return to sport, the possibility of ongoing difficulties and the long-term consequences of injury. By addressing these factors early in the rehabilitation journey, it may prevent barriers from occurring later.

Consistent with previous literature, kinesiophobia and fear of re-injury were significant barriers to completing rehabilitation and returning to sport [15]. Kinesiophobia is not associated with strength and power and must not be assumed from physical function [16]. Kinesiophobia is particularly evident during the late phase of rehabilitation as jumping, landing and change of direction exercises are reminiscent of the injury mechanism. Participants should be supported by a well-trained physiotherapist to safely expose patients to psychologically stressful situations to alleviate fears of re-injury [17]. Patients may also be lacking a formal period of training before return to sport to help refamiliarise themselves with their sport [17], which is associated with a reduced risk of re-injury [18].

In some patients’ referral to a sports psychologist may be appropriate.

Several factors influenced motivation to participate in rehabilitation. Common factors included the lengthy rehabilitation process, competing life demands, time commitment, rising costs, setbacks, or plateaus in rehabilitation. As self-motivation has been linked to home exercise completion [5], ongoing support is crucial to educate patients on the importance of completing rehabilitation and utilising goal setting to foster self-efficacy in rehabilitation [19]. Further external motivation can be gained by drawing from the success of others who have returned to sport, such as professional athletes.

### Physiological

ACL reconstruction may result in a vicious cycle of injury, sedentary lifestyle, weight gain and increased risk of musculoskeletal injury and cardiovascular disease [20]. Impairments after injury and surgery and fear of re-injury may reduce physical activity and are associated with unfavourable weight gain post-surgery [21–23]. Participants reported that weight gain significantly affected their sense of well-being and motivation. Referral to a dietician for nutritional advice with support provided by the physiotherapist for energy expenditure may be appropriate for some patients.

Consideration of the goals and return to sport aspirations of older individuals need to be respected. Despite their age, they often had the same goals and aspirations to return to sport as their younger counterparts. However, older individuals were often told not to play sport again based on their age, creating a barrier to applying themselves to rehabilitation and meeting physical benchmarks, which, regardless of age, will enhance reaching long-term function [24]. The majority of the participants had ongoing pain, which is common after ACL reconstruction [9]; as such, dosing of rehabilitation to the appropriate level to avoid persistent knee pain and the early identification of complications is essential to facilitate progression through rehabilitation and achieve successful outcomes [25].

### Rehabilitation service

Patients are seeking a plan which encompasses all phases of rehabilitation. A clear area of deficiency within the participant's rehabilitation was the lack of early physiotherapy input to prepare physically for surgery and set expectations and goals for rehabilitation [26]. This is despite literature demonstrating an effective prehabilitation program can improve post-operative outcomes [27, 28]. The clinician should also consider the best service model for each patient, with models including a fixed fee, pay as you go, telehealth, group rehabilitation or a combination. When deciding on the optimal mode of care for a patient, the patient circumstances need to be considered, including access to health care, financial restraints, exercise and rehabilitation knowledge and skills and personal preferences. Regardless of potential issues with affording ongoing care, it was typically the physiotherapist who dictated the frequency of ongoing appointments. A survey of Australian Physiotherapists reported a preference for decreasing rehabilitation frequency as rehabilitation progressed [2], posing a barrier to receiving optimal care if expectations for rehabilitation are not in alignment. For example, early in rehab, post-operative restrictions provided a significant barrier to access health care. Still, participants highly valued frequent reviews for advice, education, and prescription of exercises combined with hands-on therapy. The mid-phase of rehabilitation is characterised by patients regaining function, with some highly valuing ongoing frequent care.

In contrast, others feel they did not need frequent reviews and felt prepared to continue working towards their mid-stage goals independently. In the later phases, participants are often finding it hard to complete rehabilitation due to lack of confidence, technical exercise prescription knowledge, failure of the physiotherapist to progress the rehabilitation to sports-specific activities and rising costs. In combination with the competing demands of work and family, it potentially results in early cessation of supervised rehabilitation.

In our cohort, most participants either failed to complete rehabilitation or did not participate in any form of sports-specific rehabilitation, which is consistent with previous literature [4]. All participants who ceased rehabilitation early wished they had continued supervised rehabilitation for longer to achieve better long-term function, in line with rehabilitation guidelines which promote 9–12 months of rehabilitation [1, 5, 29]. Increased supervision during the late phase may increase the likelihood of participants overcoming physical and mental barriers to return to sport [17, 30]. While research in group rehabilitation is limited, it was supported by participants. It may provide a cost-effective mode to achieve higher supervision and progress rehabilitation to build the physical capacities and skills required for return to sport. Previous literature has demonstrated that interaction with others provides shared experience and enhances motivation, confidence, accountability, and encouragement [31].

### Rehabilitation characteristics

Effective exercise prescription to achieve appropriate functional levels before return to sport is an essential component of a successful ACL reconstruction rehabilitation [6]. As demonstrated in this theme, effective exercise prescription entails more than the appropriate set and repetition scheme but must also consider the contextual factors of the patient and the mode of exercise delivery [5, 32]. The exercise prescription process starts with setting a clear set of goals collaboratively with the patient to enhance engagement, shared understanding, and compliance with rehabilitation. While the exercise type has been shown not to influence adherence in chronic musculoskeletal problems, it appears paramount that clinicians consider the patient's circumstances, such as access to equipment and exercise knowledge, to remove barriers to exercise [33, 34]. Videos of exercises could also increase the accuracy of completion of independent exercise programs [33]. Paired with effective coaching utilising clear non-technical instructions and external focused cues, it will improve independent technical proficiency, exercise understanding, and adherence. While evidence for goal setting improving physical and psychosocial outcomes after musculoskeletal injury is of low quality at present, participants reported it as a valuable strategy during their care [19]. Combining appropriate exercise delivery and goal setting with regular communication and supportive information also provides a valuable opportunity to enhance the therapeutic relationship, which is positively correlated with patient outcomes [35].

Finally, despite recent evidence questioning the validity of return to sport tests [36, 37], a formal return to sport assessment offers excellent value to patients to identify physical deficits and psychological readiness. An appropriate assessment will also provide information to patients on areas they need to continue to work on to avoid ongoing pain and problems with the knee. Formal return to sport testing presents a significant opportunity for clinicians to improve the return to sport decision-making process and influence long-term ACL reconstruction outcomes [6].

### Interactions with others

The initial interactions with the surgeon play a role in preparing for rehabilitation. Facilitating a supportive environment builds confidence and motivation for rehabilitation and cohesive messaging across the treatment team. Negative interactions with family and friends, team and coaches, and the media may create barriers to rehabilitation. On the other hand, supportive family and team environments that facilitated rehabilitation by encouraging exercise completion, allowing ongoing involvement with the team, and drawing motivation from public sports figures enhanced their rehabilitation compliance.

### Limitations

There are several limitations to the current study. The identified factors are a representation of the perceptions and opinions of the individuals involved in the research. These may not be able to be extrapolated or be representative of a different population or context. The identified themes were unable to be weighted; as such, we cannot determine the strength of one of them over another to influence a patient’s rehabilitation. The cohort included a wide range of participants, as such perspectives may differ in a more homogeneous population based on age, gender or return to sport goals. On average, participants were 6.6 years post ACL reconstruction introducing a recall bias due to the relatively long period since active rehabilitation. The analysis and coding for themes were completed by one researcher, influencing the trustworthiness of the thematic analysis. Finally, the semi-structured interview guide and coding were guided by a-priori themes based on previous literature, creating bias within the coding structure. Some participants also had a prior relationship with the researchers. Future research should confirm these factors, explore the weighting of these factors, and investigate the effect of implementing a tailored rehabilitation program addressing the barriers and promoting rehabilitation facilitators on patient outcomes.

## Conclusion

There are many reasons why patients may fail to adhere to or participate in rehabilitation, which may be a key factor in high re-injury and low return to sport rates after ACL reconstruction. This qualitative investigation identified key aspects of a patient’s rehabilitation in which they encounter a variety of barriers and facilitators of ACL reconstruction rehabilitation. These aspects, such as psychological and physiological factors, the rehabilitation service, rehabilitation characteristics, and interactions with others, were consistently identified by this cohort as factors that affected their rehabilitation. The themes may provide targets for clinicians to improve rehabilitation. However, the themes must be evaluated in future trials to assess whether interventions to remove barriers or enhance facilitators improves subsequent outcomes such as return to sport and re-injury rates.

## Supplementary Information

**Additional file 1**. Focus group semi-structured interview guide, the semi-structured interview guide used in each focus group as referenced on line 98 of the manuscript.

**Additional file 2**. Themes and additional verbatim quotes, additional verbatim quotes from each of the identified themes and subthemes as referenced on line 148 of the manuscript.

## Data Availability

The datasets used and analysed during the current study are available from the corresponding author on reasonable request.
