# Supplementary material for: ‘Such a massive part of rehab is between the ears’; barriers to and facilitators of anterior cruciate ligament reconstruction rehabilitation: a qualitative focus group analysis
Source: BMC Sports Sci Med Rehabil. 2022 Jun 15;14:106. doi: 10.1186/s13102-022-00499-x (PMC9199234; doi:10.1186/s13102-022-00499-x)
Supplement: Supplementary file 1 — Additional file 1. Focus group semi-structured interview guide, the semi-structured interview guide used in each focus group as referenced on line 98 of the manuscript. [file 13102_2022_499_MOESM1_ESM.docx]

**Focus Group Semi Structured Interview Guide**

**Official welcome**

This is an initial study into your experiences of ACL rehabilitation. Not much has been done on this before, so we are excited to hear what you have to say as patients.

In this focus group; ideally, we'd just like you all to talk to each other about your experiences. To try and get your views of what's important and not ours, we want to take as little part in this as possible. A few points we want to emphasise are:

- Speak clearly and not softly with only one person talking at a time. It will make it very hard to hear the tape recorder clearly.
- It is important for us to hear everyone’s ideas and opinions. There are no right or wrong answers to questions – just ideas, experiences and opinions, all are valuable.
- It is important for us to hear all sides of an issue – both positive and negative.
- It is important that you also ask each other questions and speak to each other

We have some broad questions we will ask to get the ball rolling or steer things back on course if the discussion goes too far off track. You can say as much or as little as you want; we might try and come in on the conversation if it seems that someone isn't getting a chance to chip in. It will take about an hour; though if some or all of you want to run on a bit, that's fine.

The conversation's being tape-recorded for us to transcribe and look at later. One of us will be taking notes; this is just to make it easier to know who's talking when we're listening back.

Do you have any questions? You can always get back to us later.

**Interview Guide**

Generic probing questions

- Can you tell me more?
- Could you explain what you mean?
- What was that like for you?
- Do you mean….
- Does anyone feel/experience the same/differently?

**Interview** **questions**

1. **Intro:** What is your name, injury history and sport of choice?
2. **Expectations and satisfaction:** Before surgery, what goals/expectations did you have about your rehabilitation and were you satisfied with the outcome?
   1. Prompt: Were you hoping to return to your preinjury level of activity? Did you return to your preinjury level? Why?
   2. Prompt: Did the amount of time it took to complete rehabilitation seem longer or shorter than what you expected?
   3. Prompt: What did you think would be involved?
   4. Prompt: Why are you not satisfied? With the outcome or with the rehabilitation?
   5. Prompt: Do you have any ongoing problems with your knee? Why?
3. **Frequency and duration:** What determined how frequently and how long you attended physiotherapy/rehabilitation for?
   1. Prompt: Did your physiotherapist dictate your appointments? Or was it your surgeon? Or yourself?
   2. Prompt: Do you feel like you received care frequently enough and for long enough? Did that change through the phases of rehab?
   3. Prompt: Did the cost or insurance coverage limit therapy visits?
4. **Barriers:** Tell me what you thought about the rehabilitation you were asked to complete. What made it hard to complete?
   1. Prompt: What changed as you progressed through rehabilitation?
   2. Prompt: When you were given home exercises, were they provided in a way that you were able to do the same things independently? If not, what could we have done to make them easier to complete at home?
   3. Prompt: Do you think the exercises were boring, progressed to slowly or were not sport specific? Did that affect your chance of completing them?
   4. Prompt: Did you have any difficulty getting motivated to complete exercise throughout the rehabilitation process?
   5. Prompt: Was it hard to find time to complete rehab or attend sessions?
   6. Prompt: Were there physical limitations (pain, strength, swelling)?
   7. Prompt: Were you concerned about performing them correctly and safely?
   8. Prompt: Did you perform any of your rehabilitation as part of a group or with supervision? What did you like/dislike about this?
5. **Fear:** How did fear of re-injury affect your rehabilitation?
   1. Prompt: Were you ever afraid to participate for fear of getting hurt again?
   2. Prompt: Did rehabilitation help reduce your fears of or improve your confidence in participating in activities after your surgery?
6. **Facilitators:** What other factors would have been helpful to you during your rehabilitation after ACL reconstruction?
   1. Prompt: Did your relationship with your physiotherapist affect your rehabilitation?
   2. Prompt: Was there enough guidance provided for you through your time in rehabilitation? If not, what would have helped?
   3. Prompt: Did you complete a regular re-assessment or goal setting?
   4. Prompt: What role did your friends, family or team members play in your rehabilitation?
   5. Prompt: Did you have access to any digital or electronic resources such as apps or websites? Did you find them helpful?
   6. Prompt: Do you see a role for online telehealth rehabilitation?
7. **Program design:** If you were in charge of developing a rehabilitation program after ACL reconstruction, what would it include?
8. **Final call:** Can you think of anything we should have talked about but didn’t?

Interview script guided by Paterno et al. (2019).
