# Supplementary material for: ‘Such a massive part of rehab is between the ears’; barriers to and facilitators of anterior cruciate ligament reconstruction rehabilitation: a qualitative focus group analysis
Source: BMC Sports Sci Med Rehabil. 2022 Jun 15;14:106. doi: 10.1186/s13102-022-00499-x (PMC9199234; doi:10.1186/s13102-022-00499-x)
Supplement: Supplementary file 2 — Additional file 2. Themes and additional verbatim quotes, additional verbatim quotes from each of the identified themes and subthemes as referenced on line 148 of the manuscript. [file 13102_2022_499_MOESM2_ESM.docx]

**Additional Table. Thematic analysis and exemplar quotations**

| **Organising theme 1: Psychological** | | | | | | |
| --- | --- | --- | --- | --- | --- | --- |
| **Subtheme** | **Exemplar quotations** | | | | | |
| 1.1 Expectations^a^ | I had a decent idea of what it entailed probably in terms of the time length, but probably not in terms of the actual commitment to rehab. I knew it would be nine to 12 months, but I didn't realise how much I'd actually have to do. *(Participant 16)* | | | | | |
|  | I think that's the thing, they say to you, oh, you'll be fine, get back to playing at the same level that you do. And you do, but you play the same level that you did, but you'll come home and be in pain, or you'll just have weird days where you wake up and your knee's feeling a bit niggly, or just things like that, that no one actually told you about. *(Participant 10)* | | | | | |
| 1.2 Kinesio-phobia and fear of reinjury^a^ | I remember we were trying to get me to jump single, like land single leg, and jumping diagonally. I just, my head couldn't do it. I'd be on the step, and I'd sort of go one, two. I couldn't do it…. *(Participant 2)* | | | | | |
|  | I think fear is a really good motivator during the rehab process, just fear of re-injuring it again, obviously. It made me want to complete my rehab. *(Participant 18)* | | | | | |
| 1.3 Motivation^a^ | Sometimes it was just a bit of motivation because I'd just done enough like I was traumatised, I was recovering from the surgery, I'd just had enough pain, and I got to a certain point when I was just out of that, and I probably should've kept going with it. *(Participant 8)* | | | | | |
|  | Oh, she just did her ACL a year ago, and now she's back paying that same level." It gave me more motivation to keep going; it was like, "I can get back to that level and become better than what I was as long as I keep working. *(Participant 12)* | | | | | |
| 1.4 Support | The point of going to a group of people together is because you go home and speak to your partner, and she goes, oh, I don't know what's wrong with you. You've only done your knee. Whereas if you go and speak to someone that actually have an understanding, which that mental side of it, that in itself can't be taken away from how that improves your ability to get better. *(Participant 1)* | | | | | |
|  | I never really had any access to a support group, but I think that probably could be beneficial because there's lots of frustrations that you have during your rehab that you can kind of share and get that support from a group who are experiencing it at the same time, potentially. *(Participant 17)* | | | | | |
| **Organising theme 2: Physiological** | | | | | | |
| **Subtheme** | | **Exemplar quotations** | | | |  |
| 2.1 Age | | I was 42, and I thought I wanted to get back to competitive touch, but I haven't done that either... Yeah, so I probably, in hindsight was a little bit unrealistic about the impact of the surgery at that age. *(Participant 7)* | | | |  |
|  |  | I think because I was so young, they wanted to make sure that I could continue to play whatever sport I wanted for the rest of my life. *(Participant 5)* | | | |  |
| 2.2 Weight gain | | I didn't realize how much I was eating until I stopped being active, because then the weight piles on because I was going to the gym five, six times a week until my injury slowed down. (*Participant 6)* | | | |  |
|  |  | I think maybe having a discussion with the physio early on about things that you can do to burn off energy. I found that I had so much energy in my body and no release for it, especially in the early days. Like for me to concentrate at work, I need to release the energy. I just had no idea how to. *(Participant 15)* | | | |  |
| 2.3 Pain^a^ | | The cyclops lesion was probably affecting that, because of the anterior knee pain, and I couldn't do, stepping down was very hard. *(Participant 6)* | | | |  |
|  |  | It was the pain; I don't want to go in and do something that's painful again when all my friends are out there enjoying this. *(Participant 10)* | | | |  |
| **Organising theme 3: Rehabilitation service** | | | | | |  |
| **Subtheme** | | | **Exemplar quotations** |  |  |  |
| 3.1 Initial service delivery | | | Went to the doctors and they said that they didn't think anything was wrong with it and then went to the physio and they're like, yeah, you've done your ACL. Went and got the scans done, it was ACL, MCL, LCL, and meniscus. *(Participant 14)* |  |  |  |
|  |  |  | I even wonder if for some people, like for me, personally, had I have gone to my physio, and they said this is not a three-month process, this is a nine-month process, and we want to book you in for a nine-month period or however long, it's almost like a contract that you've entered into with that physio, and then you've got a relationship that there's a bit of accountability. *(Participant 2)* |  |  |  |
| 3.2 Access to health care^a^ | | | I couldn't drive, I couldn't get myself there, so I was relying on other people getting me to the physio, so that would probably be... That one would be a major one. *(Participant 9)* |  |  |  |
|  |  |  | Then of course, you need to travel to physio and everything like that. When I did my first one, it would take me a really long time to get to and from but if some of that could've been done over a phone call it would've been much more convenient. *(Participant 18)* |  |  |  |
| 3.3 Frequency of and duration of service^a^ | | | I could feel the strength, but once I had to try and do alternate jumping, and jumping longer distances, running, jumping, and also doing it on my own because financially, you run out of visits, and yeah. Back to work, all of that stuff, I just went aw, and I probably didn't really finish it. *(Participant 2)* |  |  |  |
|  |  |  | I think probably early input's important and then there's a period where you'd probably get a little bit less input, but I think return to sport's pretty important, which I didn't do any of, ended up with subsequent injuries, I guess, returning to sport. *(Participant 16)* |  |  |  |
| 3.4 Group rehabilitation^a^ | | | I mean that group thing when you get to that stage where you're kind of incremental if you can then go and almost share it with a group of people and motivate each other going along then that there would've been excellent. *(Participant 1)* |  |  |  |
|  |  |  | That was a key thing I found in it, motivation. Everyone had the same injury, everyone just wanted to get back where they were, and most people were semi-professional, so it was pretty competitive just every day there. *(Participant 3)* |  |  |  |
| 3.5 Telehealth^a^ | | | If there was a combination, I think it would work, because there's definitely a time in the rehab where I needed hands-on therapy, but there were definitely times where that option would've sufficed plenty. *(Participant 17)* |  |  |  |
|  |  |  | If you're doing the exact same thing, but you're just progressing with weight or reps, then that can be done easily over FaceTime or a phone call or whatever you want to use, kind of thing. I think it can be beneficial, especially financially as well, maybe. *(Participant 18)* |  |  |  |
| **Organising theme 4: Rehabilitation characteristics** | | | |  |  |  |
| **Sub-Theme** | | **Exemplar quotations** | | | |  |
| 4.1 Exercise delivery | | Yeah, my app was really good. I found it so useful to have and just to step through ... Like, just having a sheet of exercises, only I could actually watch the demonstration of the exercise, especially if I wasn't familiar with it because you can be shown by a physio, then you've got to try and remember that and replicate it in a gym. *(Participant 17)* | | | |  |
|  |  | You can use this, or you can do this because it has exactly the same outcome. So, I didn't have to use equipment, I could go to the gym if I wanted to, but all the stuff I could do was at home with stretch bands, or pillows, or bags of rice. He gave me multiple options to do mine, because I was, during rehab, I could do them pretty much anywhere. *(Participant 12)* | | | |  |
| 4.2 Informational support^a^ | | It's about the information around recovery, and It's a whole ton of resources there, but it's about having something that you can trust, and the issue again is it doesn't matter what the program is, everyone will have an exception to the program, and because we're all individuals. *(Participant 4)* | | | |  |
|  |  | I was watching quite a few Americans who made little videos about their journey, and they'll kind of snapshot you through, so I kind of looked through. What were they like at three months, and six months? I was trying to get a vision what the journey to expect. *(Participant 7)* | | | |  |
| 4.3 Goal setting and reassessment^a^ | | And going back to the physio each week, it was like I was proud to share my results with someone and then him to acknowledge that as well, and especially over such a long period of time that you spend with them. *(Participant 15)* | | | |  |
|  |  | You've got in front of you a 10K, so now you've got to actually make sure you get to that point. So, I think things like that helped me along the way and my physio and I would work on like, what new challenges are we going to pop in there that we're going to reach? *(Participant 10)* | | | |  |
| 4.4 Therapeutic relationship^a^ | | I wish there were a little bit more information about the selection of who you choose as your physio for the whole prehab, surgery, rehab. I didn't see that first person again because I didn't feel it was useful and then I went into a proper real sports clinic where they were working with AFL players, and I think that was really helpful. *(Participant 10)* | | | |  |
|  |  | But for me it would've been more around just having more of a mentor throughout the process in some way, even if it was every two weeks to check in to say, "How are you doing? Keep going, you're going well. Where are you up to?" Just because I didn't have a regular physio, so for me it would've been great to have just had someone to keep you focused on the next goal and the next goal. *(Participant 8)* | | | |  |
| **Organising theme 5: Interactions with others** | | | | | |  |
| **Subtheme** | | | **Exemplar quotations** | |  |  |
| 5.1 The surgeon | | | Going into [surgery] beforehand saying, there's no rush for the surgery. Or letting them know that, oh, here's a program that you need to do before you go to the surgery just to make sure that it's going to help with your recovery. Would be great. I reckon I probably would've trained harder than what I did beforehand if I knew it was going to help get me back on the field. *(Participant 9)* | |  |  |
|  |  |  | The surgeon makes a difference… the first guy who was we can get you back, we can get you back, and that was his whole message, and this is who I've got back, and if you do what I say, you'll be fine. *(Participant 4)* | |  |  |
| 5.2 Family and friends^a^ | | | I couldn't get out of the house and whatever, but you work out who your friends are and all that kind of stuff by who's coming around to actually pick you up and then drive you places. *(Participant 9)* | |  |  |
|  |  |  | One of my best mates, he joined me halfway through my rehab, and we did the exercises together, and I think that really helped to keep pushing me because to keep going and to better myself every week. *(Participant 15)* | |  |  |
| 5.3 Team and coaches^a^ | | | I didn't hang around any of my team because ... I watched the game. I felt, not guilty, watching the game, but it aggravated me knowing that I could be doing better than people on the court or knowing that I could be out there doing a good job. *(Participant 15)* | |  |  |
|  |  |  | I think keep involved with the sport, with your teammates, and coaching. Like I went to training sessions to just help out with the equipment and stuff, and just keep that chemistry with your teammates, it was a big motivation for me just to get it done. *(Participant 13)* | |  |  |

^a^ denotes a-priori theme (5), ACL = anterior cruciate ligament, ACL, MCL = medial collateral ligament, LCL = lateral collateral ligament
